# Improved Transcatheter aortic valve implantation for aortic regurgitation using a new-type stent: the first preclinical experience

**DOI:** 10.1186/s13019-020-01327-4

**Published:** 2020-09-29

**Authors:** Tong Kan, Lan Gu, Hongjiang Lu, Junxiong Cao, Zhijun Zhu, Danning Wu, Zhidong Zhu, Xiang Chen

**Affiliations:** 1 Department of Cardiology, Changhai Hospital, Second Military Medical University, Shanghai, 200433 China; 2 Department of Cardiology, ShangRao Hospital of Traditional Chinese Medicine, Shangrao, 334000 Jiangxi China; 3 Department of medical image, The 903 Hospital of the Chinese People’s Liberation Army, Hangzhou, 310004 Zhejiang China; 4 Department of Cardiology, The 903 Hospital of the Chinese People’s Liberation Army, No. 40 JiChang Road, Jianggang District, Hangzhou, 310004 Zhejiang Province China

**Keywords:** Aortic valve replacement, Aortic regurgitation, Stent shifting

## Abstract

**Background:**

In this study, we sought to evaluate the feasibility of improved transcatheter aortic valve implantation (TAVI) in noncalcified aortic valve by using the novel concept of double-layer ChenValve prosthesis. TAVI was initially considered as an alternative treatment for high-risk patients with aortic stenosis. However, non noncalcified aortic valve disease was considered as a contraindication to TAVI.

**Methods:**

ChenValve prosthesis, which consisted of a self-expanding Nitinol ring, a balloon-expandable cobalt-chromium alloy stent and a biological valve, was implanted at the desired position under fluoroscopic guidance in a transapical approach through a 20F sheath in 10 goats. Aortic angiography was performed to measure the diameter of the aotic annulus and assess the performance of the artificial valve. The ultrasound was used to evaluate the regurgitation or paravalvular leakage and trans-prosthetic vascular flow velocity postoperatively. The aortogram and transthoracic echocardiography were applied to observe whether the valve stent was implanted at the desired position.

**Results:**

ChenValve prosthesis was successfully transppical implanted in all animals. The aortogram and transthoracic echocardiography performed immediately after implantation revealed that the valve stent was implanted at the desired position. There was no significant paravalvular leakage, obstruction of coronary artery ostia, stent malpositioning or dislodgement occurred.

**Conclusions:**

This preliminary trial with the novel double-layer ChenValve prosthesis demonstrated the feasibility of improved TAVI in noncalcified aortic valve. The mechanism of Nitinol ring-guided locating the aortic sinus enables us to anatomically correct position the artifact valve. This improved strategy seems to make the TAVI process more safe and repeatable in noncalcified aortic valve.

## Background

Aortic valve disease is common among elderly individuals, and its prevalence increases with age [1]. Transcatheter aortic valve implantation (TAVI) is developed as the standard of care for inoperable patients with severe symptomatic calcific aortic stenosis and is recommended by clinical practice guidelines [2, 3]. However, TAVI is rendered less effective in patients with pure aortic valve regurgitation. The standard of care for these patients remains surgical aortic valve replacement [4, 5]. The main reason is that the noncalcified aortic valve lacks fluoroscopic landmarks and anchor site for prosthesis, which tends to increase the risk of prosthesis dislocation in TAVI [6, 7]. In addition, absent or minimal calcification of aortic valve induced insufficient anchoring results in prosthesis dislodgement, which can lead to poor prognosis. For these reasons, improving the TAVI is critical for the therapy of AR.

The Edwards HELIO transcatheter aortic dock is a novel approach for the treatment of NAVR and this method uses a pre-placed dock behind the aortic leaflets to facilitate implantation of a SAPIEN XT valve [8]. However, the whole procedure is too complicated to be popularized in clinic practice. Another technique is to use two pigtail catheters to place in two coronary sinuses [9]. This technique could significantly reduce the valve stent dislocation in TAVI using CoreValve prosthesis for AR patients. However, the follow-up residual aortic regurtitation requires to implant a second valve [9]. A small single-centered series demonstrated the feasibility of transapical TAVI with the new generation self-expandable ACURATE TA device (Symetis SA, Ecublens, Switzerland) in high-risk patients with severe AR [10]. However, the size of delivery system of AVURATE device was too large (28F) for transapical TAVI, which would cause additional injuries to cardiac muscle. The new generation of Jena-Valve prosthesis (JenaValve Tchnology GmbH, Munich, Germany) is featured with a unique clip fixation mechanism of the native aortic valve leaflets that might offer anchorage for the valve stent during TAVI for AR [11]. The JenaValve was proved to be a promising therapeutic strategy [12] and becomes the only TAVI device approved for the treatment of high-risk or inoperable patients with severe AR.

In this study, we developed the ChenValve with a novel double-layer stent and featured with a nitinol outer-ring. This unique design could ensure the accurate position of the aortic sinus and provide an anchor for prosthesis to improve the success rate of TAVI in noncalcified aortic valve. The purpose of the study was to evaluate the feasibility of transapical TAVI by the using ChenValve in a goat model.

## Methods

### Aortic valve stent and delivery device design

**Fig. 1:**
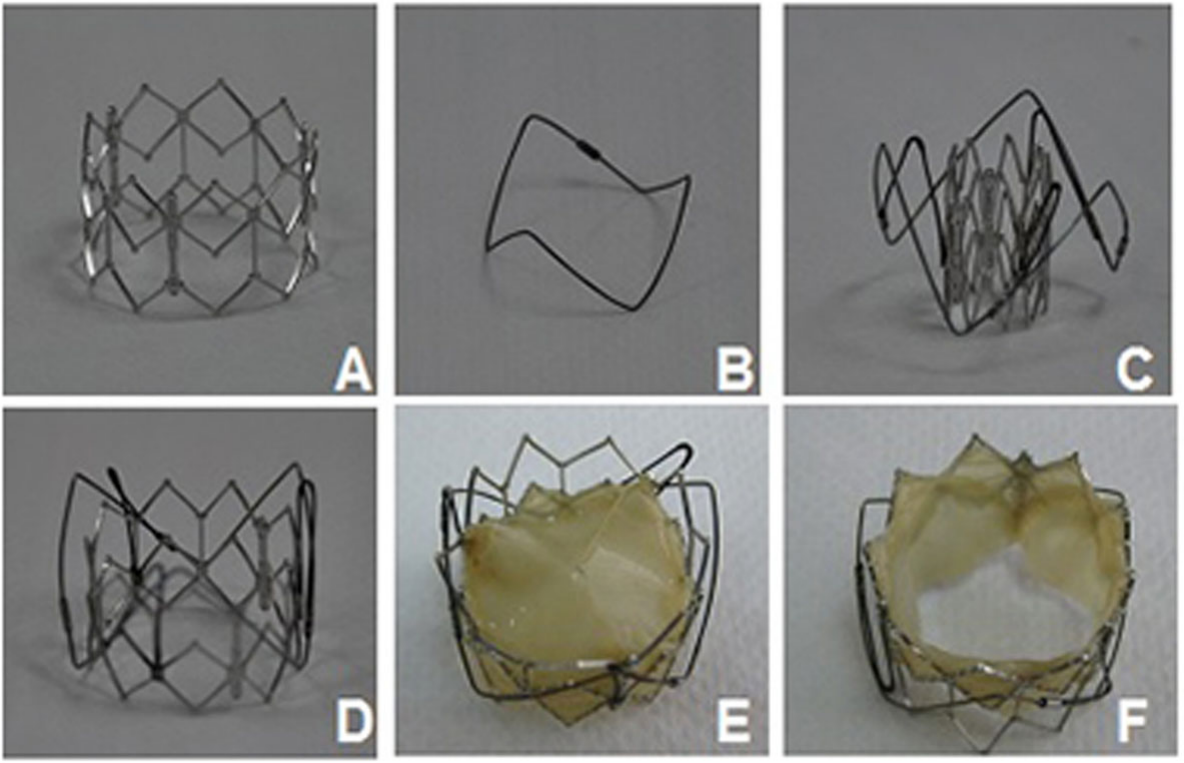
Prosthetic aortic valved stent. **a** balloon-expandable cobalt-chromium alloy stent. **b** self-expanding nitinol ring; **c**. compressed stent; **d**. aortic stent; **e**, **f**. ChenValve system)

The ChenValve prosthesis and delivery device was designed for transapical aortic valve implantation and was made by LePu Medical Technology (Beijing) Co.,Ltd.. The ChenValve prosthesis (Fig. 1) consisted of a balloon-expandable cobalt-chromium alloy stent, a self-expanding nitinol ring and a biological valve. Nitinol ring was designed for three “feelers” to be placed into the three aortic sinuses to achieve an anatomically correct position of aortic valve annulus. The stent was made of cobalt-chromium alloy by laser cutting and had an overall length of 18 mm. Two parts of this device were connected by three V-shape Nitinol memory alloy threads. Three leaflets cut from tissue-fixated porcine pericardium were mounted on the stent. The additional polyester skirt sealed the cells of the cobalt-chromium alloy stent frame to prevent paravalvular leaks. The diameter of Nitinol ring was designed to be 3 mm less than that of the stent. The prothesis was available in sizes of 20, 23, and 26 mm, according to the diameter of the stent. All sizes could be loaded into a 20F delivery system for implantation.

**Fig. 2:**
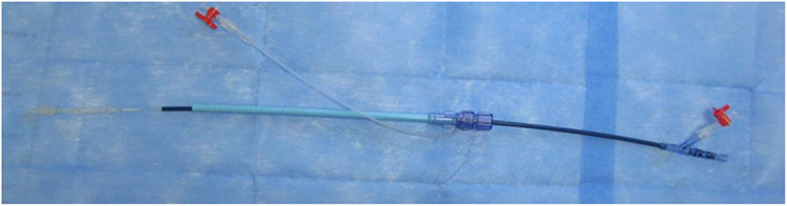
Delivery system of stent

The stent delivery system was comprised of an outer sheath (20F) and a stent delivery catheter which formed an integral unit (Fig. 2). The proximal segment of a stent delivery catheter was an inflatable balloon. The valved stent was compressed on the balloon and then be withdrawn into the outer sheath by retracting the delivery catheter. The tip of the delivery catheter had a conical smooth transition made of silica gel material to allow implantation of the delivery catheter by performing apical puncturing. The delivery system allowed a two-step release of the prosthesis: first, releasing the Nitinol ring to locate the aortic annulus, and then releasing the cobalt-chromium alloy stent by injecting the balloon with diluted contrast agent once achieved a correct positioning. According to the size of the balloons, the stent delivery system was classified into 20, 23, and 26 mm sizes, respectively.

### Experimental animals

Ten healthy goats (5 male and 5 female), with an average weight of 22.3 ± 1.85 kg, were obtained from the Marine Animal Medical Research Institute. Animal studies were approved by the Experimental Animal Ethics Committee of Zhongshan Hospital (Approval no.20170426181252). All animals received humane care in compliance with the Guide for the Care and Use of Laboratory Animals (www.nap.edu/catalog/5140.html). Each goat was anesthetized by intramuscular injection of ketamine (10-mg/kg) after 8 h of fasting, followed by intravenous injection of propofol (0.2 mg·kg^− 1^·min^− 1^). The airway was maintained by endotracheal intubation and a respirator.

### Valve stent implantation

**Fig. 3:**
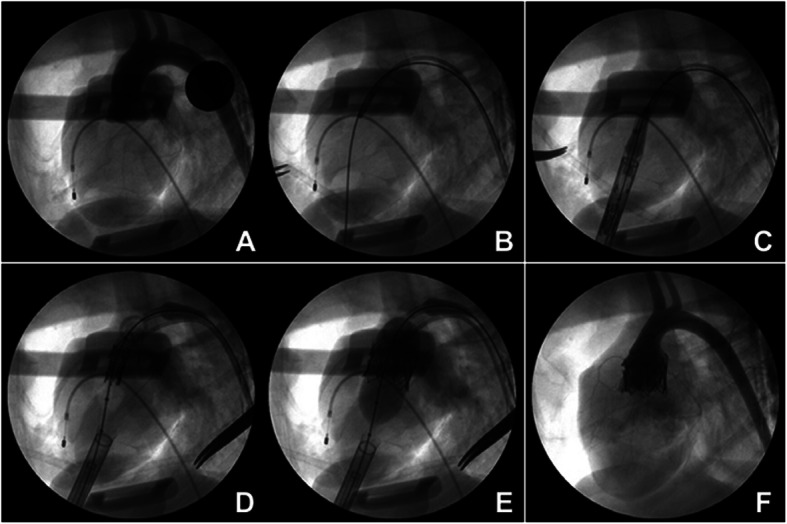
Transcatheter aortic valve implantation (TAVI) by using a double-layer ChenValve prosthesis. (**a**. Aortic angiography were performed to measure the diameter of the aortic annulus; **b**. A stiff guidewire was inserted and across the aortic valve into the descending aorta; **c**. Delivery device was inserted into LV from the heart apex; **d**. Three “fellers” of Outer-ring of Prosthesis was taken to locate the three aortic sinus; **e**. Prosthesis was expanded and released; **f**. The performance of the artificial valve was assessed by aortic angiography)

The electrocardiogram and oxygen saturation were monitored throughout the entire operation. The right femoral artery and femoral vein were punctured and inserted with a 6-Fr leak-proof sheath. Heparin 50 U/kg IV was administered. The temporary pacing lead was introduced into the apex of the right ventricle via femoral vein sheath. A 6-Fr pigtail catheter was delivered via femoral artery sheath. Aortic angiographic procedures were performed to determine the best imaging position and the diameter of the aortic annulus was measured (Fig. 3a). The valve stent size should be suitable to the annulus diameter, and then was compressed to the balloon of the delivery catheter by using a crimping machine. The diameter of the selected stent was 2–4 mm larger than the that of the animal aortic annulus. Transapical access was achieved through a left anterolateral minithoracotomy in the fourth intercostals space and purse-string sutures with 4–0 Prolene were applied to the left ventricular apex. The apex of the left ventricle was punctured, and then a stiff guidewire was inserted and acrossed the aortic valve into the descending aorta under fluoroscopic guidance (Fig. 3b). A 20-Fr delivery system with the ChenValve prosthesis was inserted into the left ventricle from the heart apex over the stiff guidewire (Fig. 3c).

The delivery catheter was fixed when prosthesis had advanced into a supra-annular position under fluoroscopic guidance. Then the outer sheath was withdrawn slowly, and the Nitinol alloy outer-ring of prosthesis automatically expanded. Then the operator located the three positioning “fellers” of Nitinol ring in the corresponding three aortic sinuses on fluoroscopy, thereby embracing the native leaflets (Fig. 3d). After correct position of three “fellers” was verified in 2 different fluoroscopic angulations. Ventricular temporary rapid pacing (250–300 beats/min) was used to slow down the velocity of blood flow from the left ventricle and reduced the movement amplitudes of aortic annulus. Then the prosthesis was expanded and precise released in ideal location by injecting the balloon with a contrast agent diluted 5:1 (Fig. 3e).

The contrast agent was extracted when the valve stent was fully opened. Temporary pacing was shut down, then the delivery catheter and guidewire were retraced from the heart and the apex closed with the purse string suture. Subsequently, the position of the valve stent and performance of the artificial valve were assessed by aortic angiography (Fig. 3f).

**Fig. 4:**
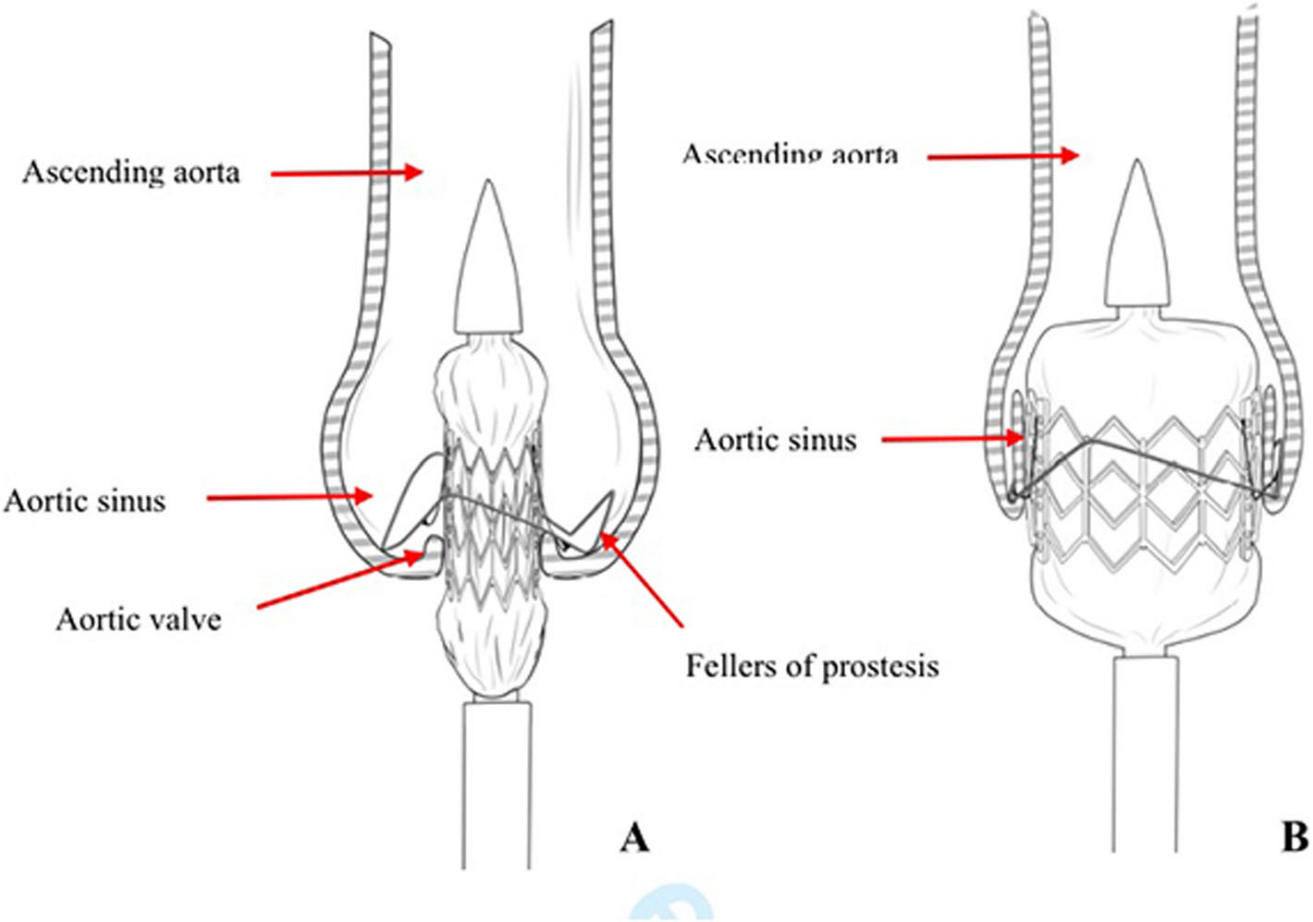
Process of Valve Stent Implantation (**a**. Three “fellers” of Outer-ring of Prosthesis was taken to locate the three aortic sinuses; **b**. Prosthesis was expanded and released)

The diagram of process of Valve Stent Implantation was shown in the Fig. 4.

### Postoperative treatment

The chest was closed if no active bleeding occured in the thoracic cavity. After the sheaths of the femoral artery and vein were removed, the hemostasis of femoral artery was performed by applying direct pressure to puncture points for 20 min. Propofol was not used until 15 min before the end of the operation. After the operation, 0.5-mg atropine and 1-mg neostigmine were intravenously injected to reverse muscle relaxation, and 160–320 million units of penicillin were administered to prevent postoperative infection. The tracheal catheter of the experimental goat was removed when autonomous respiration and blood pressure were restored. Penicillin was administered for 7 days to prevent infection, and then low-molecular-weight heparin and aspirin were administered for 3 and 90 days, respectively. The wound was disinfected daily with iodine. 2 weeks later, the sutures were removed.

### Assessment of valve stent position and functionality

In each case, the location and function of the prosthetic aortic valve were detected by aortic root angiography and transthoracic echocardiography immediately after the operation. One randomly selected goat was euthanized two hours after successful implantation for macroscopic inspection at necropsy. The general health status of goat models, including eating, defecation, and activities, were observed. The performance of the prosthetic aortic valve, including perivalvular leakage and aortic regurgitation, was evaluated by transthoracic echocardiography 1 month after operation.

### Statistical analyses

All the statistical analyses were performed using the SPSS 18.0 software package. The measurement data were indicated as mean ± SD. The data from the different groups were analyzed using a repeated-measures method. *p* < 0.05 was considered statistically significant.

## Results

**Fig. 5:**
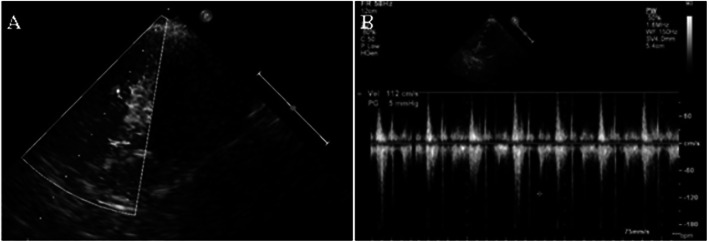
Postoperative Ultrasound (**a**. No regurgitation or paravalvular leakage was found; **b**. trans-prosthetic valvular flow velocity was normal)

**Fig. 6:**
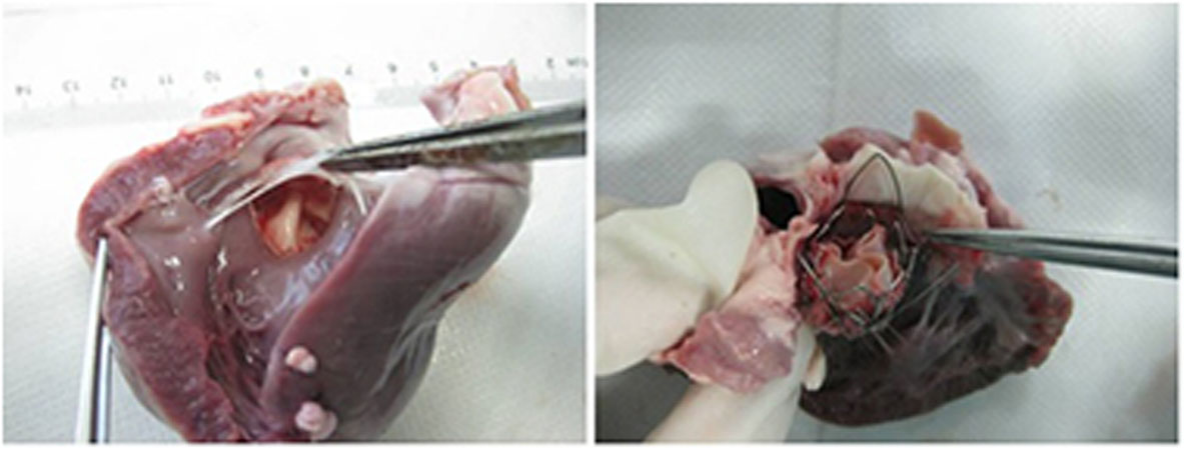
Anatomical examination of heart after TAVI(**a**. valve stent was well “anchored” at the aortic wall of desired position; **b**. Three “fellers” of Outer-ring of Prosthesis was taken to locate the three aortic sinus)

The deployment of prostheses for all 10 goats were successfully completed. The mean diameter of the valve stent was 21.18 ± 1.28 mm. The operation duration and X-ray exposure time were 128.90 ± 10.67 min and 14.40 ± 3.44 min, respectively (Table 1). In each case, the results from aortogram and transthoracic echocardiography (Fig. 5) that were applied immediately after implantation revealed that the valve stent was implanted at the desired position, There were no obstruction of coronary artery ostia, malpositioning or stent dislodgement occurred. No moderate--gross regurgitation or paravalvular leakage were observed. One goat (No. 6) was sacrificed 2 h after successful implantation to observe the position and function of the valve. The cardiac anatomy of the sacrificed animal showed that the valve stent was well “anchored” on the aortic wall of desired position (Fig. 6). Three “fellers” of Outer-ring of Prosthesis were taken to locate the three aortic sinus. The upper edge of the valve stent was lowered from coronary artery ostia by 3-5 mm, while the lower edge of the valve stent was far away from the mitral valve. The functions of the coronary artery and mitral valve were not affected. The remaining 9 goats survived for more than 1 month with normal diet and activity. Transthoracic color Doppler ultrasound performed 1 month after operation showed normal trans-prosthetic vascular flow velocity and normal position of the prosthetic valve without stenosis, insufficiency apparent and stent dislodgement.

**Table 1:** Data of the operational experiment animals

No	Body mass (kg)	Aortic ring diameter (mm)	Stent diameter (mm)	Operation time (min)	Radiographic time (min)
1	28.2	23.1	26	142	19
2	29.3	22.8	26	138	17
3	28.4	21.5	26	145	20
4	27.5	20.8	23	133	16
5	28.1	20.8	23	128	15
6	25.2	19.2	20	124	13
7	26.9	19.8	23	118	11
8	27.5	20.2	23	129	14
9	28.3	22.3	26	113	10
10	29.1	21.3	26	119	11
Mean	27.85	21.18	24.20	128.90	14.60
SD	1.18	1.28	2.10	10.67	3.44

## Discussion

There has been emerged some devices for TAVI. The Edwards HELIO transcatheter aortic dock used a pre-placed dock behind the aortic leaflets to facilitate implantation of a SAPIEN XT valve [8]. CoreValve prosthesis had two pigtail catheters to place in two coronary sinuses to significantly reduce the valve stent dislocation in TAVI for AR patients [9]. The ACURATE TA device (Symetis SA, Ecublens, Switzerland) is used for high-risk patients with severe AR, however, the delivery system was too large (28F) for transapical TAVI [10]. The Jena-Valve prosthesis (JenaValve Tchnology GmbH, Munich, Germany) offered anchorage for the valve stent during TAVI for AR [11] and is a promising therapeutic strategy [12] for high-risk or inoperable patients with severe AR.

However, all these devices have their limitations. The Edwards HELIO transcatheter aortic dock is novel approach for the treatment of NAVR, which uses a pre-placed dock behind the aortic leaflets to facilitate implantation of a SAPIEN XT valve [8]. But the whole procedure is too complicated to generalize this technique in clinic practice. Another technique for placing pigtail catheters in two coronary sinuses was useful to decrease valve stent dislocation in TAVI using CoreValve prosthesis for AR patients [9]. The result of operation was not ideal, and the residual aortic regurtitation required implantation of a second valve in 8 of 43 patients due to residual AR. A small single-centered series demonstrates the feasibility of transapical TAVI with the new generation self-expandable ACURATE TA device (Symetis SA, Ecublens, Switzerland) in high-risk patients with severe AR [10]. However, the size of delivery system of AVURATE device is too large (28F) for transapical TAVI and would cause more damage of cardiac muscle. The new generation of Jena-Valve prosthesis (JenaValve Tchnology GmbH, Munich, Germany) features a unique clip fixation mechanism of the native aortic valve leaflets that may offer anchorage of the valve stent during TAVI for AR [11]. The JenaValve had demonstrated promising results [12] . However, Jena-Valve prosthesis is only a implanted transapical approach.

**Fig. 7:**
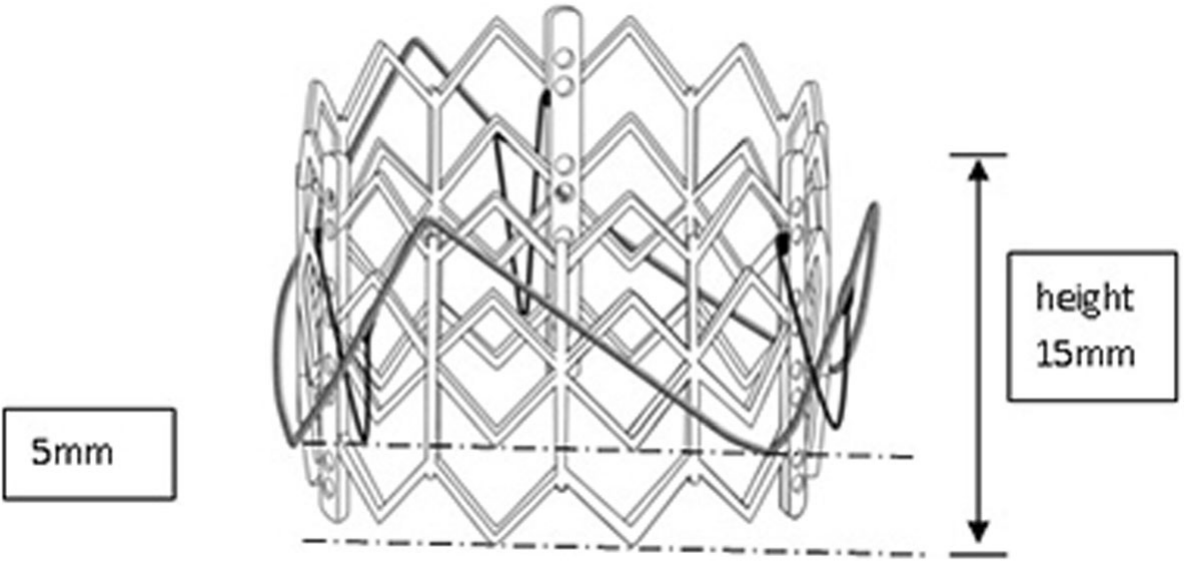
The bottom edges of stent is designed 5 mm lower than the bottom edges out-ring

**Fig. 8:**
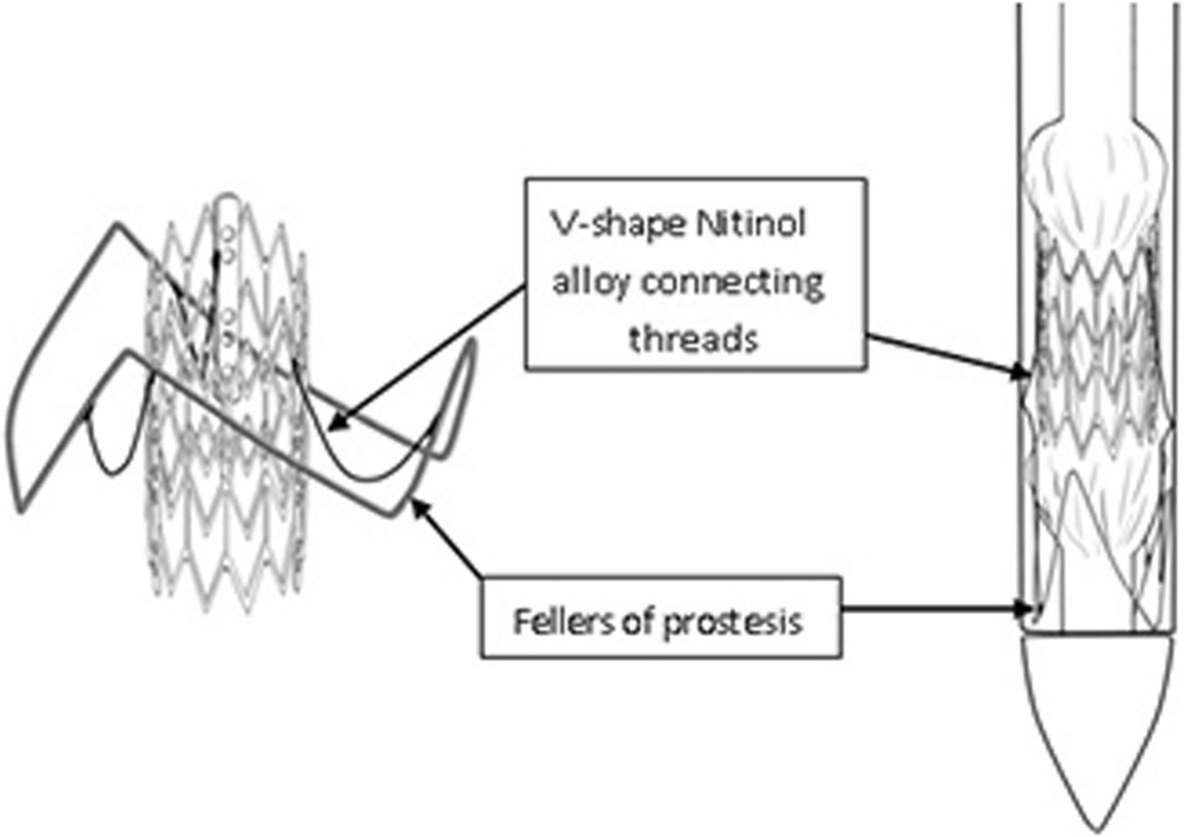
The three V-shape Nitinol alloy connecting with three threads

The present study aimed to evaluate the feasibility of ChenValve prosthesis, which consists of a self-expanding nitinol ring and a balloon-expandable cobalt-chromium alloy stent. The mechanism of Nitinol ring-guided locating the aortic sinus allows for anatomically oriented implantation of stent in noncalcified aortic valves. Not only can the correct position of Nitinol ring be verified in fluoroscopic angulations, but also the tactile feedback (movement of the delivery catheter once the three “feeler” against the three aortic sinus) can guide the operator for optimal deployment. The height of the ring is 10 mm, and the upper edge of ring and stent are at the same level. After the three “feelers” of rings are placed at the three aortic sinuses, the correct position of the prothesis below the coronary ostia was determined. The bottom edges of stent are designed 5 mm lower than the bottom edges out-ring (Fig. 7), which allows the bottom of stent to be precisely implantated 5 mm lower than the aortic sinus. This design can effectively reduce the risk of atrioventricular block. Compared with the JenaValve, the main body of ChenValve is a balloon-expanding cobalt-chromium alloy stent. The advantage of balloon-expanding stent is that the length is shorter than that of the self-expanding stent, and a smaller part of the stent is prominent to the left ventricular outflow tract. This design can effectively abrogate the occurrence of atrioventricular block. The diameter of ring is 2–3 mm smaller than that of the stent. Once the stent is expanded, the stent can be deployed within the outer-ring to sandwich the native aortic leaflets in between, in favor of prosthesis stabilization and reducing the occurrence of dislodgement and perivalvular leakage. Another distinctive design, the three V-shape Nitinol alloy connecting threads would be straightened into “一” shape, so that the ring would be stretched above the stent after prothesis is compressed (Fig. 8), which allows the profile of prothesis to become much smaller, in favor of delivery with smaller sheath (20F). The design of the skirt is intended to prevent paravalvular. In out study, no mass or moderate perivalvular leakage were observed.

Before the chen-valve was expanded, ventricular temporary rapid pacing (250–300 beats/min) was used to slow down the velocity of blood flow from the left ventricle and reduce the dynamic amplitudes in the aorta, to ensure that the implantation position of prosthesis is accurate. The Nitinol alloy outer-ring of prosthesis is not a solid anchoring structure. In addition to looking at the image, the precise positioning also depends on the hand feeling of the operator during prosthesis positioning.

Many ARs are frequently related to the dilation of aortic root. Dilation of sinuses and sino-tubular junction may be an intense obstacle for TAVI. In this study, it has not been proven whether the chen-valve is suitable for AR, since dilation of aortic root is not proved in this research.

According to the characteristics of the chen-valve, it is possible to implant the Chen valve through the transfemoral route, and we will confirm this hypothesis in further studies.

Our study is the first report to evaluate the composite ChenValve prosthesis in vivo test. The ChenValve prosthesis is only available for implantation via the transapical approach. This study still has limitations. First, the number of study animals is small and a larger study samples are needed to confirmed the results. In addition, the experimental animals are healthy goats instead of AR model. Moreover, the patients with AR have a more complex and variable anatomy.

## Conclusion

This initial trial using the novel double-layer ChenValve prosthesis demonstrated the feasibility of of TAVI in noncalcified aortic valve. The mechanism of Nitinol ring-guided locating of the aortic sinus enables anatomically corrent positioning and seems to make the TAVI process safe and reproducible in noncalcified aortic valve.

## Data Availability

All data generated or analyzed during this study are included in this published article.

## Abbreviation

TAVI: Transcatheter aortic valve implantation
